# Relationship Between Serum Total Carbon Dioxide Concentration and Bicarbonate Concentration in Patients Undergoing Peritoneal Dialysis

**DOI:** 10.7759/cureus.14119

**Published:** 2021-03-25

**Authors:** Keiji Hirai, Susumu Ookawara, Momoko Matsuyama, Taisuke Kitano, Kiyonori Ito, Yuichiro Ueda, Tatsuro Watano, Shinji Fujino, Kiyoka Omoto, Yoshiyuki Morishita

**Affiliations:** 1 Division of Nephrology, First Department of Integrated Medicine, Saitama Medical Center, Jichi Medical University, Saitama, JPN; 2 Department of Laboratory Medicine, Saitama Medical Center, Jichi Medical University, Saitama, JPN

**Keywords:** serum total carbon dioxide, bicarbonate, peritoneal dialysis

## Abstract

Background

Few studies have assessed the relationship between serum total carbon dioxide (CO_2_) and bicarbonate ion (HCO_3_^−^) concentration in patients undergoing peritoneal dialysis. We determined the agreement between serum total CO_2 _and HCO_3_^− ^concentration and the diagnostic accuracy of serum total CO_2_ for the prediction of low (HCO_3_^−^ <24 mEq/L) and high (HCO_3_^−^ ≥24 mEq/L) bicarbonate concentrations in patients on peritoneal dialysis.

Methods

We collected 245 samples of venous blood from 51 patients on peritoneal dialysis. Independent factors that correlated with the HCO_3_^−^ concentration were analyzed using multiple linear regression analysis. The diagnostic accuracy of serum total CO_2_ was evaluated by receiver operating characteristic (ROC) curve analysis and a 2×2 table. Agreement between serum total CO_2_ and HCO_3_^−^ concentration was assessed by Bland-Altman analysis.

Results

Serum total CO_2_ was independently correlated with HCO_3_^−^ concentration (β = 0.354, *p* < 0.001). The area under the curve of serum total CO_2_ for the identification of low and high bicarbonate concentrations was 0.909. The diagnostic accuracy of serum total CO_2_ for the prediction of low and high bicarbonate concentrations was: sensitivity, 91.5%; specificity, 74.7%; positive predictive value, 53.5%; negative predictive value, 96.5%; and accuracy, 78.8%. Bland-Altman analysis showed a moderate agreement between serum total CO_2_ and HCO_3_^−^ concentration.

Conclusion

Serum total CO_2_ correlated closely with the HCO_3_^−^ concentration in patients undergoing peritoneal dialysis. Serum total CO_2_ might be useful for predicting low and high bicarbonate in peritoneal dialysis patients.

## Introduction

Metabolic acidosis is a commonly observed complication in patients with chronic kidney disease (CKD), including those undergoing peritoneal dialysis, and is associated with bone mineral loss, protein energy-wasting, insulin resistance, and higher mortality risk [1-4]. It also contributes to a rapid decline in residual renal function [5]. Therefore, early detection and accurate diagnosis of metabolic acidosis is important to preserve residual renal function and improve prognosis in patients undergoing peritoneal dialysis.

In Japan, blood-gas analyzers are available in most hospitals. Therefore, bicarbonate ion (HCO_3_^−^) measured using arterial/venous blood gas samples has been widely used for the assessment of metabolic acidosis in peritoneal dialysis patients [6]. A lower HCO_3_^−^ concentration has been reported to be associated with increased mortality in patients undergoing peritoneal dialysis [7]. Because the HCO_3_^−^ concentration is an important predictor of mortality, a specific device measurement and syringe are necessary, in addition to the blood samples used for blood-gas analyses [8].

The serum total carbon dioxide (CO_2_) concentration represents the total amount of carbon dioxide in the serum. It can be readily measured, along with creatinine, urea, and electrolytes, using a biochemical analyzer in clinical settings [9]. Furthermore, serum total CO_2_ has been shown to be correlated strongly with the HCO_3_^−^ concentration in both patients with CKD not undergoing renal replacement therapy [10] and patients undergoing hemodialysis [11]. However, few studies have investigated the relationship between serum total CO_2_ and the HCO_3_^−^ concentration in patients undergoing peritoneal dialysis. We analyzed the relationship between serum total CO_2_ and the HCO_3_^−^ concentration in peritoneal dialysis patients.

## Materials and methods

Ethics approval

The study was approved by the ethics committee of Saitama Medical Center, Jichi Medical University (S17-052), and was conducted according to the principles contained within the Declaration of Helsinki. The requirement of informed consent was waived and an opt-out method was used because of the retrospective design of the study.

Participants

Inclusion criteria were: (i) age >20 years; (ii) CKD stage G5D; (iii) regular peritoneal dialysis; (iv) simultaneous measurement of serum total CO_2 _and HCO_3_^− ^concentrations. Exclusion criteria were: (i) hemodialysis and (ii) renal transplantation.

Study design

This was a single-center, retrospective, cross-sectional study. We analyzed the patient data obtained from medical records from the Division of Nephrology, Saitama Medical Center, between April 2017 and March 2019. The laboratory data of blood tests and venous blood-gas tests obtained simultaneously were used for analyses. The relationship between serum total CO_2 _and the HCO_3_^− ^concentration was analyzed using Pearson’s correlation coefficient. Independent factors correlated with the HCO_3_^− ^concentration were analyzed using multiple linear regression analysis. The diagnostic accuracy of serum total CO_2_ for low and high bicarbonate was analyzed using receiver operating characteristic (ROC) curve analysis and a 2×2 table. The correlation between serum total CO_2_ and HCO_3_^−^ concentration was analyzed using Bland-Altman analysis.

Laboratory methods

Blood and urinary parameters were determined by the Department of Clinical Laboratory, Saitama Medical Center. Samples of venous blood were collected in EDTA-containing tubes from the antecubital vein in all patients and centrifuged within 15 minutes to obtain serum. Serum total CO_2_ was measured within 15 minutes after centrifugation using an automated biochemical analyzer (JCA-BM6070; JEOL, Tokyo, Japan), as were biochemical parameters (hemoglobin, total protein, serum albumin, blood urea nitrogen, serum creatinine, sodium, potassium, chloride, calcium, phosphate, magnesium, and glucose). Serum total CO_2_ was determined by an enzymatic method using a commercial kit (Toyobo, Osaka, Japan) in an automated biochemical analyzer. Total weekly urea clearance (Kt/V) was measured by calculating the sum of the residual renal and peritoneal clearances of urea and converting this to a weekly value [12]. Residual renal urea clearance was determined using 24-hour urine urea divided by plasma urea concentration. Total body water volume was estimated from height, weight, age, and gender using Watson's formula [13].

Samples of venous blood for gas analyses were collected in a heparinized blood-gas syringe from the brachial vein simultaneously with samples for other blood tests and analyzed within 10 minutes to obtain values for pH and the partial pressure of carbon dioxide (pCO_2_). The pH and pCO_2_ of blood were measured using a blood-gas analyzer (Rapidlab-1265; Siemens Healthcare Diagnostics, Tarrytown, New York). The HCO_3_^−^ concentration was calculated from measured pH and pCO_2_ using the Henderson-Hasselbalch equation [14]: pH = 6.1 + log([HCO_3_^−^]/pCO_2_ × 0.03).

Statistics

Statistical analyses were performed using JMP v11 (SAS Institute, Cary, North Carolina). Continuous variables were expressed as mean ± standard deviation when they were normally distributed and as median and interquartile range when non-normally distributed. Categorical variables were expressed as numbers and percentages. The peritoneal dialysis duration was not normally distributed; therefore, this variable was transformed using a natural logarithm. The relationships between two variables were evaluated using Pearson’s correlation coefficient. Linear regression analysis was used to identify parameters that independently correlated with HCO3− concentration. The parameters that significantly correlated with HCO_3_^−^ concentration in simple linear regression analyses were included in subsequent multiple linear regression analysis. The diagnostic accuracy of serum total CO_2 _was determined using ROC curve analysis and a 2×2 table. The area under the curve (AUC), sensitivity, specificity, positive predictive value, negative predictive value, and accuracy were calculated for the identification of low (HCO_3_^−^ <24 mEq/L) and high (HCO_3_^−^ ≥24 mEq/L) bicarbonate concentrations. The cut-off value for HCO_3_^−^ was set at 24 mEq/L based on a previous study [15]. Agreement between serum total CO_2_ and HCO_3_^−^ concentration was assessed using the Bland-Altman method. P < 0.05 was considered to represent statistical significance.

## Results

Patient characteristics

Patients’ characteristics and medications are shown in Table 1. A total of 245 blood samples from 51 patients (35 males and 16 females, mean age 62.3 ± 13.6 years, mean peritoneal dialysis duration 27.5 ± 29.6 months) were obtained. Forty-one patients (80.4%) were on continuous ambulatory peritoneal dialysis (CAPD), 33 patients (64.7%) on automated peritoneal dialysis (APD), and 23 patients (45.1%) on a combination of CAPD and APD. The mean total weekly Kt/V was 1.68 ± 0.39. Thirty-three percent of the patients had diabetes mellitus. The proportions of the patients receiving each medication were: corticosteroid, 3.9%; β-blocker, 43.1%; renin-angiotensin system inhibitor, 72.5%; aldosterone receptor antagonist, 7.8%; loop diuretic, 64.7%; thiazide diuretic, 39.2%; tolvaptan, 29.4%; potassium binder, 0.0%; phosphate binder, 82.4%; vitamin D analog, 54.9%; calcimimetic, 21.6%; and sodium bicarbonate 0.0%. Calcium concentrations of each peritoneal dialysis solution were as follows: icodextrin solution, 1.75 mmol/L; lactate-buffered solution, 1.25 mmol/L; and bicarbonate-buffered solution 1.25 mmol/L.

**Table 1:** Patient characteristics and medication Abbreviations: APD, automated peritoneal dialysis; CAPD, continuous ambulatory peritoneal dialysis; Kt/V, urea clearance. Valuables are shown as mean ± standard deviation, median [interquartile range], or number (%).

Number of patients		51
Number of samples		245
Age (year)		62.3 ± 13.6
Gender male (number, %)		35 (68.6)
Body mass index (kg/m^2^)		22.7 ± 3.6
Peritoneal dialysis duration (month)		18.2 [9.8-33.7]
Peritoneal dialysis modality	CAPD (number, %)	41 (80.4)
	APD (number, %)	33 (64.7)
	CAPD and APD (number, %)	23 (45.1)
Peritoneal dialysis solution	Icodextrin solution (number, %)	28 (54.9)
	Lactate-buffered solution (number, %)	14 (27.5)
	Bicarbonate-buffered solution (number, %)	37 (72.5)
Diabetes mellitus (number, %)		17 (33.3)
Corticosteroid (number, %)		2 (3.9)
β-blocker (number, %)		22 (43.1)
Renin–angiotensin system inhibitor (number, %)		37 (72.5)
Aldosterone receptor antagonist (number, %)		4 (7.8)
Loop diuretic (number, %)		33 (64.7)
Thiazide diuretic (number, %)		20 (39.2)
Tolvaptan (number, %)		15 (29.4)
Potassium binder (number, %)		0 (0.0)
Phosphate binder (number, %)		42 (82.4)
Vitamin D analogue (number, %)		28 (54.9)
Calcimimetic (number, %)		11 (21.6)
Sodium bicarbonate (number, %)		0 (0.0)
4-hour dialysate/plasma creatinine		0.65 ± 0.11
Total weekly Kt/V		1.68 ± 0.39
Renal weekly Kt/V		0.69 ± 0.43
Peritoneal weekly Kt/V		1.00 ± 0.30

Relationship between serum total CO_2_ and HCO_3_^−^ concentration

**Figure 1:**
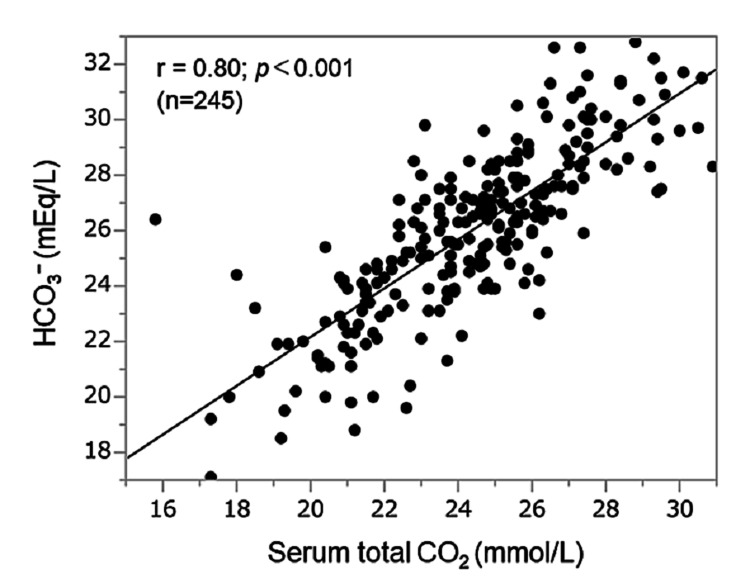
Relationship between serum total CO2 and HCO3− concentration CO2: carbon dioxide; HCO3−: bicarbonate ion

Figure 1 shows the correlation between serum total CO_2_ and HCO_3_^−^ concentration. Serum total CO_2_ was correlated with HCO_3_^−^ concentration significantly and closely (r = 0.80; p < 0.001).

Factors correlated with HCO_3_^−^ concentration

Simple linear regression analyses showed that HCO_3_^−^ concentration was significantly negatively correlated with gender male, body mass index, serum albumin, blood urea nitrogen, creatinine, uric acid, sodium, potassium, chloride, phosphate, and magnesium, and with the use of a bicarbonate-buffered solution, phosphate binder, and vitamin D analog. HCO_3_^−^ concentration was also significantly positively correlated with diabetes mellitus, four-hour dialysate/plasma creatinine, total weekly Kt/V, peritoneal weekly Kt/V, total calcium, and serum total CO_2_, and with the use of CAPD, CAPD and APD, icodextrin solution, lactate-buffered solution, loop diuretic, thiazide diuretic, and tolvaptan. Multiple linear regression analysis was performed using the variables that showed significant correlations with HCO_3_^−^ concentration in simple linear regression analyses (Table 2). This analysis revealed that total weekly Kt/V (standard coefficient [β] = 0.119, p = 0.027), serum albumin (β = -0.171, p = 0.007), blood urea nitrogen (β = -0.138, p = 0.011), sodium (β = 0.352, p < 0.001), chloride (β = -0.629, p < 0.001), total calcium (β = 0.204, p < 0.001), phosphate (β = -0.155, p = 0.006), and serum total CO_2_ (β = 0.354, p < 0.001) were independently correlated with HCO_3_^−^ concentration.

**Table 2:** Simple and multiple linear regression analyses of the parameters correlating with HCO3− concentration Abbreviations: APD, automated peritoneal dialysis; CAPD, continuous ambulatory peritoneal dialysis; CO2: carbon dioxide; HCO3−, bicarbonate ion; Kt/V, urea clearance; Log, logarithm

Parameter	Simple linear regression analysis		Multivariate linear regression analysis	
	Standard coefficient	P value	Standard coefficient	P value
Age (year)	0.083	0.20		
Gender male (yes vs. no)	-0.235	<0.001	0.084	0.09
Body mass index (kg/m^2^)	-0.194	0.002	-0.006	0.91
Log-peritoneal dialysis duration (month)	0.018	0.78		
CAPD (yes vs. no)	0.353	<0.001	0.007	0.91
APD (yes vs. no)	-0.114	0.08		
CAPD and APD (yes vs. no)	0.189	0.003	0.087	0.11
Icodextrin solution (yes vs. no)	0.242	<0.001	-0.122	0.06
Lactate-buffered solution (yes vs. no)	0.242	<0.001	0.002	0.97
Bicarbonate-buffered solution (yes vs. no)	-0.242	<0.001	0.000	---
Diabetes mellitus (yes vs. no)	0.214	<0.001	0.072	0.15
Corticosteroid (yes vs. no)	0.112	0.08		
β-blocker (yes vs. no)	0.058	0.37		
Renin-angiotensin system inhibitor (yes vs. no)	0.012	0.85		
Aldosterone receptor antagonist (yes vs. no)	0.013	0.84		
Loop diuretic (yes vs. no)	0.289	<0.001	0.052	0.43
Thiazide diuretic (yes vs. no)	0.256	<0.001	0.016	0.76
Tolvaptan (yes vs. no)	0.165	0.010	-0.070	0.22
Potassium binder (yes vs. no)	0.000	---		
Phosphate binder (yes vs. no)	-0.131	0.041	0.040	0.46
Vitamin D analog (yes vs. no)	-0.173	0.007	0.049	0.22
Calcimimetic (yes vs. no)	0.075	0.24		
Four-hour dialysate/plasma creatinine	0.293	<0.001	0.008	0.88
Total weekly Kt/V	0.138	0.031	0.119	0.027
Renal weekly Kt/V	-0.031	0.63		
Peritoneal weekly Kt/V	0.160	0.012	-0.092	0.05
Total protein (g/dL)	-0.112	0.08		
Serum albumin (g/dL)	-0.206	0.001	-0.171	0.007
Hemoglobin (g/dL)	-0.040	0.54		
Blood urea nitrogen (mg/dL)	-0.481	<0.001	-0.138	0.011
Creatinine (mg/dL)	-0.134	0.037	-0.014	0.83
Uric acid (mg/dL)	-0.295	<0.001	-0.016	0.71
Sodium (mEq/L)	-0.185	0.004	0.352	<0.001
Potassium (mEq/L)	-0.301	<0.001	0.025	0.66
Chloride (mEq/L)	-0.550	<0.001	-0.629	<0.001
Total calcium (mg/dL)	0.283	<0.001	0.204	<0.001
Phosphate (mg/dL)	-0.514	<0.001	-0.155	0.006
Magnesium (mg/dL)	-0.180	0.005	-0.014	0.76
Blood glucose (mg/dL)	0.094	0.14		
Serum total CO_2_ (mmol/L)	0.805	<0.001	0.354	<0.001

Diagnostic accuracy of serum total CO_2_ for the prediction of low and high bicarbonate concentrations

**Figure 2:**
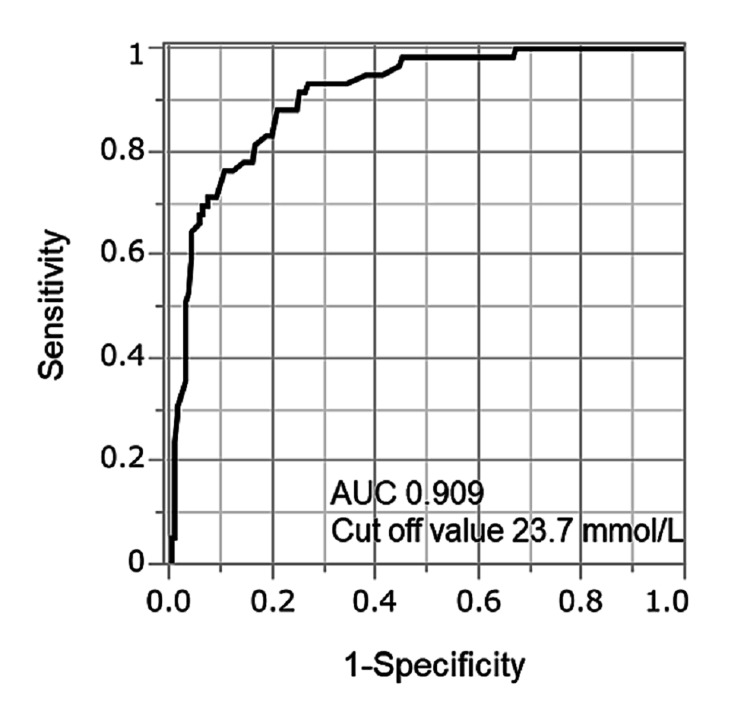
ROC curve of serum total CO2 for detecting low bicarbonate (HCO3− <24 mEq/L) and high bicarbonate (HCO3− ≥24 mEq/L) concentrations AUC: area under the curve; CO2: carbon dioxide; HCO3−: bicarbonate ion; ROC: receiver operating characteristic

The ROC curve of serum total CO_2_ for detecting low (HCO_3_^−^ <24 mEq/L) and high (HCO_3_^−^ ≥24 mEq/L) bicarbonate concentrations is shown in Figure 2. The AUC was 0.909, and the optimal cut-off value was 23.7 mmol/L. The 2×2 tables, stratified according to serum total CO_2_ and HCO_3_^−^ concentration for low and high bicarbonate, are shown in Table 3. The diagnostic accuracy measures of serum total CO_2_ for the prediction of low and high bicarbonate concentrations were as follows: sensitivity (91.5%), specificity (74.7%), positive predictive value (53.5%), negative predictive value (96.5%), accuracy (78.8%), pre-test probability (24.1%), positive post-test probability (53.5%), and negative post-test probability (3.5%).

**Table 3:** 2×2 table stratified according to serum total CO2 and HCO3− concentration for low and high bicarbonate Abbreviations: CO2, carbon dioxide; HCO3−, bicarbonate ion

		HCO_3_^-^		Total
		Low bicarbonate (HCO_3_^-^ <24 mEq/L)	High bicarbonate (HCO_3_^-^ ≥24 mEq/L)	
Serum total CO_2_	Low serum total CO_2_ (Serum total CO_2 _<24 mmol/L)	54	47	101
	High serum total CO_2_ (Serum total CO_2 _≥24 mmol/L)	5	139	144
Total		59	186	245

Correlation between serum total CO_2_ and HCO_3_^−^ concentration

**Figure 3:**
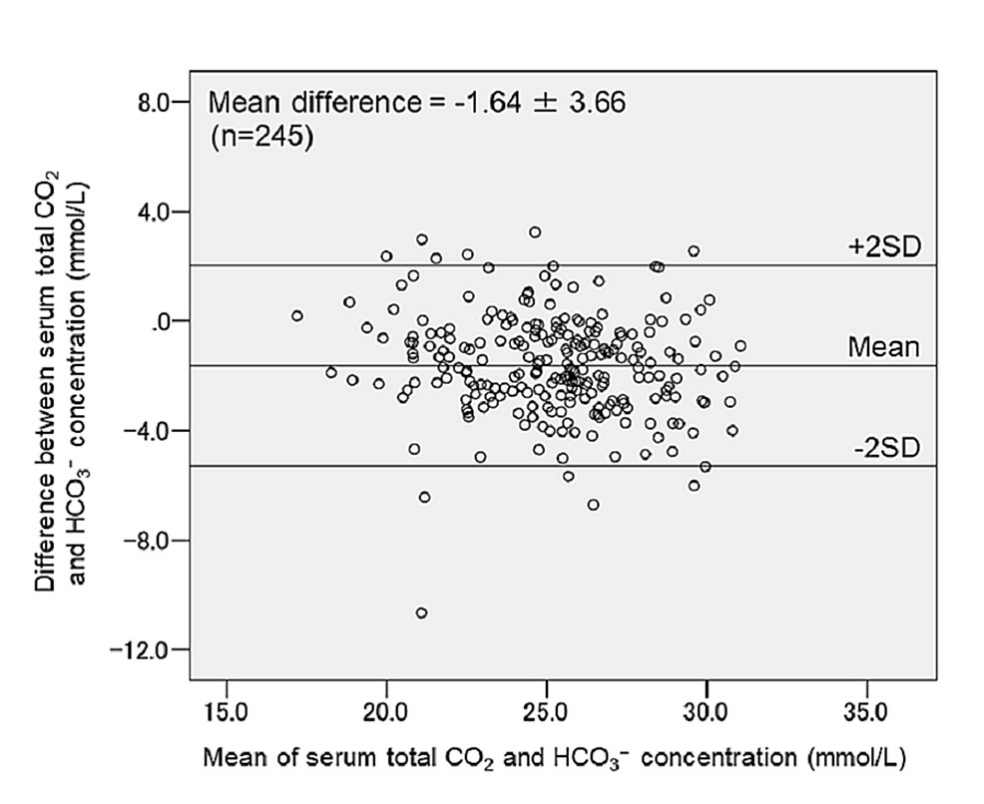
Bland-Altman plot comparing serum total CO2 and HCO3− concentration CO2: carbon dioxide; HCO3−: bicarbonate ion; SD: standard deviation

Bland-Altman analysis showed moderate agreement between serum total CO_2_ and HCO_3_^−^ concentration. The mean difference was -1.64 ± 3.66, and 95.1% of the points were included within the limits of agreement (the mean difference between the two methods ± 2 standard deviation [95% confidence interval]) (Figure 3).

## Discussion

In the present study, we investigated the relationship between serum total CO_2_ and HCO_3_^−^ concentration in peritoneal dialysis patients and found that serum total CO_2_ closely correlated with HCO_3_^˗^ concentration. We also found that serum total CO_2_ has high diagnostic accuracy for predicting low bicarbonate and high bicarbonate in peritoneal dialysis patients.

Serum total CO_2_ is a total concentration of all forms of CO_2_ in a serum sample, including HCO_3_^−^, carbonate, and dissolved CO_2_. Serum total CO_2_ value is known to be approximately equivalent to HCO_3_^−^ concentration because most of CO_2_ exists as HCO_3_^−^ in blood [9]. In fact, serum total CO_2_ has been reported to have a close correlation with HCO_3_^−^ concentration in both pre-dialysis CKD patients [10] and hemodialysis patients [11]. However, a discrepancy between serum total CO_2_ and HCO_3_^−^ concentration is sometimes observed, and temperature and acidity [16] are considered one of the causes of discrepancy in patients without renal impairment [17]. In the present study, serum albumin, calcium, chloride, sodium, phosphate, blood urea nitrogen, and total weekly Kt/V in addition to serum CO_2_ were independently correlated with HCO_3_^−^ concentration in serum.

Serum albumin represents the nutritional status of patients and is reportedly associated with dietary protein intake in peritoneal dialysis patients [18]. Protein intake is associated with metabolic acidosis because amino acids into which dietary proteins are broken down release hydrogen ions [19]. Increased serum albumin was reported to be associated with metabolic acidosis in pre-dialysis CKD patients [20]. The weak acidity of albumin has also been considered as the cause of this phenomenon [21]. These findings are consistent with our result, showing a negative correlation between serum albumin and HCO_3_^− ^concentration.

It has been reported that HCO_3_^− ^was positively correlated with calcium concentration in hemodialysis patients [22]. In the present study, HCO_3_^−^ concentration was positively correlated with calcium concentration in peritoneal dialysis patients. These findings suggest that serum HCO_3_^− ^concentration might be positively correlated with calcium concentration in patients with end-stage renal disease. There were differences in calcium concentrations among peritoneal dialysis solutions used in the present study. The possibility remains that these differences might affect the results of our study.

HCO_3_^−^ concentration has been shown to decrease along with an increase in chloride concentration through following equilibrium with HCl and NaHCO_3_: H^+^ + Cl^-^ + Na^+^ + HCO_3_^−^ = Na^+^ + Cl^-^ + H_2_CO_3 _[23]. In the present study, chloride concentration was negatively correlated with HCO_3_^−^ concentration, which is consistent with the findings of previous reports [10-11].

A cross-sectional study of peritoneal dialysis patients reported that sodium concentration was lower in patients with HCO_3_^− ^< 22 mEq/L than in patients with 22 ≤ HCO_3_^−^ < 28 mEq/L [24]. In the present study, sodium concentration was positively correlated with HCO_3_^−^ concentration. These results suggest that sodium concentration is positively associated with HCO_3_^−^ concentration in peritoneal dialysis patients.

Phosphate and blood urea nitrogen were shown to be associated with daily protein intake in patients with end-stage renal disease [25]. Protein intake is negatively associated with bicarbonate, as the amino acids into which dietary proteins are broken down release hydrogen ions [19]. Phosphate and blood urea nitrogen were reported to be negatively correlated with bicarbonate in peritoneal dialysis patients [24], which is consistent with the findings of our study.

Currently available peritoneal dialysis fluids contain alkaline anions of 35-40 mmol/L as lactate or/and bicarbonate [6]. The influx of alkaline anions from the peritoneal dialysis fluid into the blood occurs during peritoneal dialysis because the alkaline anion concentration in serum is usually lower than that in peritoneal dialysis fluid [24]. A previous study reported that dialysis adequacy assessed by daily Kt/V was positively correlated with serum bicarbonate level [26]. In the present study, the total weekly Kt/V was positively correlated with HCO_3_^−^ concentration. These results suggest that peritoneal dialysis dose-dependently increases serum HCO3− concentration caused by the influx of bicarbonate from the peritoneal dialysis fluid into the blood.

In the present study, serum total CO_2 _was closely correlated with HCO_3_^−^ concentration and showed high accuracy for the differentiation of low or high bicarbonate concentrations. Therefore, serum CO_2_ may be a good predictor of bicarbonate concentration and useful to predict whether this is low or high. However, the correlation between serum total CO_2_ and HCO_3_^−^ concentration in the present study (β = 0.323) was weaker as compared with that of hemodialysis patients (β = 0.858) [11]. The number of clinical parameters correlated with HCO_3_^−^ concentration was greater in the present study than in the previous one [11] (eight vs three), which might explain the lower correlation between serum total CO_2_ and HCO_3_^−^ in this study. The correlation between serum total CO_2_ and HCO_3_^−^ concentration might be attenuated in peritoneal dialysis patients. Further studies are necessary to confirm the close correlation between serum total CO_2_ and HCO_3_^−^ concentration and the usefulness of serum total CO_2_ for the diagnosis of low or high bicarbonate concentrations in peritoneal dialysis patients.

The measurement of serum total CO_2_ has two advantages as compared with blood-gas analyses. First, the cost of a blood gas-syringe can be saved and the amount of blood required will be reduced using serum total CO_2_ instead of a blood-gas test. Second, serum total CO_2_ can be used to predict low bicarbonate and high bicarbonate without the use of a blood-gas analyzer. Therefore, the measurement of serum total CO_2_ could reduce some of the burden on peritoneal dialysis patients and laboratory staff.

Our study had four limitations. First, it was a retrospective, observational study; therefore, selection bias could not be completely eliminated. Second, the study was performed at a single center, which limits the external validity of the results. Third, the study cohort was small, which restricts the generalizability of our findings. Fourth, we used venous blood samples for the analyses. The results might have been different if arterial blood samples had been used. Therefore, further prospective, large-scale, multicenter studies are necessary to confirm our findings.

## Conclusions

Serum total CO_2_ correlated closely with HCO_3_^−^ concentration in peritoneal dialysis patients. Serum total CO_2_ might be useful for predicting low and high bicarbonate in peritoneal dialysis patients.
